# A Fast Adaptive Multi-Scale Kernel Correlation Filter Tracker for Rigid Object

**DOI:** 10.3390/s22207812

**Published:** 2022-10-14

**Authors:** Kaiyuan Zheng, Zhiyong Zhang, Changzhen Qiu

**Affiliations:** School of Electronics and Communication Engineering, Sun Yat-sen University, Shenzhen 518107, China

**Keywords:** target tracking, kernel correlation filter, adaptive template updater, rigid object

## Abstract

The efficient and accurate tracking of a target in complex scenes has always been one of the challenges to tackle. At present, the most effective tracking algorithms are basically neural network models based on deep learning. Although such algorithms have high tracking accuracy, the huge number of parameters and computations in the network models makes it difficult for such algorithms to meet the real-time requirements under limited hardware conditions, such as embedded platforms with small size, low power consumption and limited computing power. Tracking algorithms based on a kernel correlation filter are well-known and widely applied because of their high performance and speed, but when the target is in a complex background, it still can not adapt to the target scale change and occlusion, which will lead to template drift. In this paper, a fast multi-scale kernel correlation filter tracker based on adaptive template updating is proposed for common rigid targets. We introduce a simple scale pyramid on the basis of Kernel Correlation Filtering (KCF), which can adapt to the change in target size while ensuring the speed of operation. We propose an adaptive template updater based on the Mean of Cumulative Maximum Response Values (MCMRV) to alleviate the problem of template drift effectively when occlusion occurs. Extensive experiments have demonstrated the effectiveness of our method on various datasets and significantly outperformed other state-of-the-art methods based on a kernel correlation filter.

## 1. Introduction

Target tracking is a comprehensive technology covering computer technology [1], pattern recognition, image processing [2], artificial intelligence [3] and other technologies [4]. Furthermore, target tracking technology is widely used in smart homes [5], human–computer interaction [6], virtual reality, medical diagnosis, modern military, information security [7], and other computer vision fields. Video target tracking is a technology that uses the context information of a video or image sequence to model the appearance and motion information of the target so as to predict the motion state of the target and calibrate the position [8].

Although research on video target tracking algorithms has made great progress in recent years, the effect of existing methods has not yet reached the ideal state due to the influence of the target’s appearance and size change, object occlusion, motion blur, tracking background interference and other factors. According to different tracking methods, target tracking algorithms can be divided into tracking algorithms based on correlation filtering and tracking algorithm based on deep learning. The algorithm based on a correlation filter mainly uses a correlation filter to calculate the similarities between a template image and prediction image to determine the target position in the process of tracking, while the algorithm based on deep learning mainly learns target features through training deep networks to complete video target tracking.

Bolme [9] introduced correlation filtering into video target tracking for the first time and proposed the Minimum Output Sum of Squared Error (MOSSE) algorithm. After that, the algorithm based on correlation filtering gradually became the mainstream method in the field of video target tracking.

However, with the proposal of the Alexnet [10] network, the video target tracking algorithm based on deep learning is gradually emerging, which has received wide attention in recent years and has produced many algorithms with excellent performance. Although this kind of algorithm has high tracking accuracy and strong robustness, it is difficult to run under the limited hardware conditions of an embedded platform such as DSP, FPGA or ARM, and the requirement of real-time processing is also hard to achieve because of its huge model parameters and computation. For example, SANet [11], one of the best performance target-tracking networks based on Convolutional Neural Networks (CNN), can only achieve 1FPS on a high-performance GPU NVIDIA GTX TITAN Z with 12GB of memory. Network models with strong performance achieve satisfactory accuracy and robustness at the cost of computing speed. However, in some application scenarios with high requirements on real-time performance, target-tracking algorithms based on CNNs are still difficult for engineers to consider.

The KCF [12] tracking algorithm is an excellent algorithm with high tracking speed, accuracy and robustness proposed by F. Henriques et al. As a similarity measure between two signals, a correlation filter provides us with a reliable distance measure and a reasonable interpretation scheme. However, because the algorithm uses a fixed tracking template according to the target object determined by the initial frame, it cannot deal with the problem of scale change of the target in the tracking process, and the template is easily blurred by the occluded object, leading to tracking failure. To address these challenges, this paper attempts to propose a fast multi-scale kernel correlation filter tracker with an adaptive template updater for a rigid object. In the phase of correlation filtering, we build a three-layer scale pyramid filter on the basis of KCF, make the target image of the last frame carry out correlation filtering with each layer image in the pyramid, and output the scale factor and maximum response value, which can deal with the problem of multiple scale changes of the target effectively and maintain an extremely high operating speed. In the template update phase, we propose an adaptive template updater based on the Mean of Cumulative Maximum Response Values (MCMRV) to set adaptive thresholds to limit the updating of the template, which alleviates the problem of template drift effectively when occlusion occurs. The main contributions are summarized as follows:

- A simple three-layer scale pyramid filter is embedded into KCF, which makes the tracker adapt to the scale change of the target efficiently.
- We propose an adaptive template updater based on MCMRV, which adaptively adjusts the template update threshold according to MCMRV criteria and plays a reliable role in dealing with target occlusion.
- Experimental results show that the improved algorithm can effectively solve the problems of scale variation and target occlusion in target tracking under the condition of high operation speed.

The rest of this paper is organized as follows. Section 2 presents the related work. In Section 3, we propose an improved KCF algorithm. Section 4 reports the results of the experiment. Finally, Section 5 is the conclusion of this paper and our future work.

## 2. Related Works

According to the different methods used in the observation model, target tracking algorithms can be divided into two categories, respectively, generative model and discriminant model. The discriminant model is further divided into models based on correlation filtering and deep neural networks. Generative models mainly include Kalman filter [13], particle filter [14], Meanshift [15] and Camshift [16], which are the earliest tracking models. A serious shortcoming of the generative model is that it does not update the model and always uses the model built at the beginning of the task. It does not take into account the influence of environmental changes on the target state during the task. When the target is clear in some frames, the target can be found better. However, when the target is blocked or in poor ambient light conditions, the tracking effect of this model is not satisfactory.

The discriminant model is the mainstream model of target tracking, which transforms the target tracking problem into a dichotomous problem and obtains the target by separating the target from the background through the model. This model can solve the problem of tracking targets well in complex environmental conditions, and it can be divided into two algorithms based on correlation filtering and deep learning according to the different features used.

Correlation filtering was originally used in signal processing to describe the correlation between two signals. Bolme proposed the MOSSE filter, which introduces the method of a correlation filter to track a target for the first time and has excellent performance on real-time tracking. Circulant Structure of tracking-by-detection with Kernels (CSK) [17] uses a kernel correlation filter to find the feature of the cyclic determinant of the negative sample so as to improve the tracking accuracy. KCF uses Histogram of Oriented Gradient (HOG), which replaces the pixel information of an image and obtains a large sample by the method of cyclic shift on original feature samples. A Gaussian kernel function is introduced to transform low-dimensional non-separable feature information into high-dimensional separable feature information so as to facilitate the calculation of feature correlation. Discrete Fourier transform and the properties of cyclic matrix are used to reduce the dimension of operation and improve the speed of the algorithm in the sample classifier and new sample detection. In the process of target tracking, the accuracy of the algorithm is greatly reduced due to the influence of target scale variation. Discriminative correlation filters and the exhaustive Scale Space Tracking (DSST) [18] algorithm proposed by Danelljan et al. treats target tracking as two independent problems of target center shift and scale change and trains the shift correlation filter and scale correlation filter, respectively, with a HOG feature. Later, Danelljan proposed fast DSST (fDSST) [19] on the basis of DSST and improved the performance of the algorithm by 6.13% and the FPS by 83.37% through feature reduction and interpolation. In 2015, Danelljan et al., once again, proposed the improved correlation filtering tracking algorithm Spatially Regularized Discriminative Correlation Filters (SRDCF) [20]. Its idea is to expand the search area and restrict the effective scope of the filter template to solve the boundary effect, but its running speed is obviously reduced. Background-Aware Correlation Filters (BACF) [21] proposed by H Kiani extend the HOG feature of a single channel to the HOG feature of multiple channels and uses the ADMM method to speed-up the computing speed.

The above algorithms based on improved correlation filtering can solve the problem of target scaling well, but they still update the model even when the target is blocked, which leads to the introduction of a large amount of irrelevant information into the filter. The tracking effect will be reduced if the target is blocked for a long time, and the calculation complexity is high and the amount of calculation is large. In an embedded system with limited computing speed, the real-time performance of the tracking algorithm is greatly affected.

In the task of target tracking, acquiring target features is a key problem, and deep learning has shown its powerful feature extraction and expression ability in other fields, so deep learning has been applied to the field of target tracking. Currently, commonly used neural network models include Alex, Vgg [22], ResNet [23], Yolo [24] and GAN [25].

Reference [26] proposes MDNet, a deep-learning tracking algorithm based on classification, which uses the small VGG network, and the authors think that there are common characteristics between the target in the different training videos. Therefore, they adopt multi-domain training, but the algorithm does not perform well in terms of speed and target occlusion. In reference [27], the GAN network is added on the basis of MDNet, and positive samples under occlusion are generated through the GAN network so that the classifier can deal with the problem of occlusion. However, the rapid increase in computation reduces the speed further. Reference [28] proposed a twin neural network Siam-FC, which regards tracking as solving the similarity problem and adopts two Alex networks to form a double-branch structure network. Although the running speed is improved, it can meet the real-time requirements only on the premise of using a high-performance graphics card to accelerate the operation.

The purpose of this study is to provide a tracking algorithm with excellent performance and speed for common rigid targets in engineering practice, and deep-learning-based algorithms are still difficult to fully apply in embedded platforms, so this paper will focus on tracking algorithms based on kernel correlation filtering. In this paper, an adaptive multi-scale pyramid and adaptive mean updater are used to improve the tracking performance of KCF for rigid targets.

## 3. The Proposed Approach

**Figure 1:**
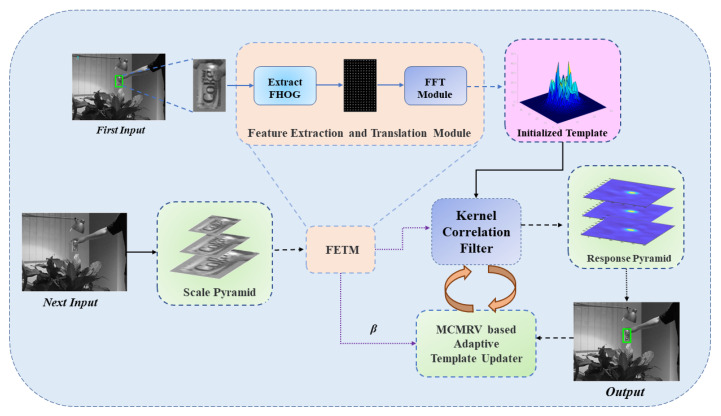
An illustration of the proposed MMKCF. We build two simple pyramid modules based on the KCF algorithm framework, namely scale pyramid and response pyramid. The two modules are embedded in the input and output of the filter, respectively. At the same time, the MCMRV-based adaptive template updater automatically monitors the response status of the filter, determines whether the target is blocked and dynamically adjusts its threshold.

Our tracker framework can be summarized as shown in Figure 1. Based on the KCF algorithm, we build a simple scale pyramid module, which can construct a multi-layer pyramid according to the target position as the input of the correlation filter. Accordingly, the filter outputs a multi-layer response pyramid from which the most suitable response value is taken as the tracking result. Considering that the response value will decrease sharply when rigid target occlusion occurs, we introduce an adaptive template updater based on MCMRV. In the process of tracking the target, the template updater adaptively adjusts the threshold according to the response results and judges whether the target occlusion occurs so as to avoid the template being polluted by noise in the process of tracking.

### 3.1. Kernel Correlation Filter Algorithm

Our approach is improved on the basis of the KCF algorithm. The KCF target tracking algorithm firstly extracts HOG features from the image information of the target region and then trains the target classifier by generating a large number of samples through cyclic displacement. A Gaussian kernel function is used to calculate the correlation response between the target sample and the sample to be tested, and the coordinate of the maximum point of the response value is the latest position of the target. Using discrete Fourier transform to transform the above process from the time domain to frequency domain can greatly reduce the amount of computation and improve the speed of computation. Finally, the classifier is updated with new target features.

Assuming that the one-dimensional vector a of 1 × *q* is the information of the target sample, the displacement matrix *P* is used to carry out cyclic displacement of the sample as $\left\{aP^{i}∥i=0,1,\ldots ,q-1\right\}$, and the matrix *P* is shown as follows:

(1) $$P=\left(\begin{array}{ccc} \begin{array}{cc} 0 & 0\\ \begin{array}{c} 1\\ 0 \end{array} & \begin{array}{c} 0\\ 1 \end{array} \end{array} & \ldots & \begin{array}{cc} 0 & 1\\ \begin{array}{c} 0\\ 0 \end{array} & \begin{array}{c} 0\\ 0 \end{array} \end{array}\\ \vdots & \ddots & \vdots \\ \begin{array}{cc} 0 & 0 \end{array} & \cdots & \begin{array}{cc} 1 & 0 \end{array} \end{array}\right)$$

$P^{i}$ represents the displacement of sample *a* by *i* bit, and $\left\{A_{i}=aP^{i},\forall i=0,1,\ldots ,q-1\right\}$ is denoted as the sample after cyclic displacement, from which $A_{i}$ can form the sample cyclic matrix as $A=[A_{0},A_{1},\ldots ,A_{\left(q-1\right)}]$.

### 3.2. Features Extraction and Regularization

The KCF algorithm is an extension of the CSK algorithm, which uses a multi-channel HOG feature instead of a gray feature to enrich the types of target sample information collection and improve the target tracking accuracy. The computed HOG features are 3×nOrients+5 dimensional. There are 2×nOrients contrast sensitive orientation channels, nOrients contrast insensitive orientation channels, 4 texture channels and 1 all zeros channel (used as a ‘truncation’ feature [29]). Using the standard value of nOrients=9 gives a 32-dimensional feature vector at each cell. This variant of HOG, referred to as FHOG, has been shown to achieve superior performance to the original HOG features.

The KCF algorithm introduces a kernel function to solve the problem of low-dimensional linear inseparability of samples and uses ridge regression to train the classifier. The classifier $f\left(X_{ji}\right)=\left(\omega ,\varphi \left(X_{ji}\right)\right)$ is trained by a minimum regularization function, φ(X) being a function that maps the sample to the Hilbert feature space. The optimal ω is obtained to minimize the function value, and the mathematical formula is expressed as follows:

(2) $$\min_{\omega }\sum_{i}^{m-1}\sum_{j}^{n-1}{\left(f\left(X_{ji}\right)-Y_{ji}\right)}^{2}+\lambda ∥\omega ∥$$

The similarity between sample *X* and $X^{\prime }$ is expressed by a Gaussian kernel function, and the following formula can be derived as follows, where $F^{-1}$ represents the Fourier transform and $\hat{X}$ is the Fourier transform of *X*:

(3) $$\kappa \left(X,X^{\prime }\right)=\exp\left(-\frac{1}{\sigma^{2}}\left(∥X^{2}∥+∥{X^{\prime }}^{2}∥-2{\left(F^{-1}\left(\hat{X}\cdot \hat{X^{\prime }}\right)\right)}^{T}\right)\right)$$

The kernel matrix $K^{X}$ constructed by training sample *X*, which can be obtained by a Gaussian kernel function. The optimal solution can be obtained through Equation (2) as follows:

(4) $$\omega =\sum_{i=0}^{m-1}\sum_{j=0}^{n-1}\alpha_{ji}\varphi \left(X_{ji}\right)$$

For the newly input sample *z*, the sample set *Z* can be obtained through feature extraction and cyclic displacement, which constructs the kernel matrix $K^{Z}$ with the training sample *X* and satisfies the cyclic conditions. From this, the response of the test sample can be obtained, and the coordinate of the point with the maximum response value represents the latest position of the target. Then the tracker updates the template parameter $\hat{a}$ and the sample parameter *X* by the following formula:

(5) $$\left\{\begin{array}{c} {\hat{a}}_{t+1}=\left(1-\mu \right){\hat{a}}_{t}+\mu {\hat{a}}_{t}\\ X_{t+1}=\left(1-\mu \right)X_{t}+\mu X_{t} \end{array}\right.$$

where ${\hat{a}}_{t+1}$ and $X_{t+1}$ are model parameters and sample parameters applied to the next frame, which are obtained from ${\hat{a}}_{t}$ and $X_{t}$ of the previous frame. μ is the template update rate. The traditional KCF algorithm still updates the template and sample parameters when the target is blocked and cannot adjust the detection region.

### 3.3. Dimension Reduction

The tracking speed based on the kernel correlation filter is determined by the calculation of the Fourier transform. In this paper, our adaptive dimension reduction strategy adopted the Principal Analysis Component Analysis (PCA) method [30]. The implementation principle will be briefly described below.

Let $x_{t}$ be a d-dimensional training sample, where each eigenvector is n-dimensional. Based on the above description, we update the template to:

(6) $$a_{t+1}=\left(1-\mu \right)a_{t}+\mu x_{t}\;$$

By minimizing the reconstruction error of $a_{t}$, the projection matrix $P_{t}$ is obtained:

(7) $$\tau =\sum_{n}∥a_{t}\left(n\right)-{P_{t}}^{T}P_{t}a_{t}\left(n\right){∥}^{2}$$

Because the reconstruction error $a_{t}$ can be minimized under the constraints of ${P_{t}}^{T}P_{t}$, our projection matrix $P_{t}$ can be calculated by the eigenvalue decomposition of the autocorrelation matrix, which corresponds to the maximum eigenvalue. We use a compressed sample and transform template to obtain the response of test sample $Z_{t}$:

(8) $$\hat{y}=F^{-1}\left(k^{a_{t-1}Z_{t}}*{\hat{a}}_{t}\right)$$

where $Z_{t}$ represents the compressed transformation template composed of HOG features.

### 3.4. Adaptive Multi-Scale Pyramid

In the process of target tracking, target scale changes often occur, but the size of the target tracking window in the traditional KCF algorithm is fixed. When the visual distance of the target changes or the camera moves, the proportion of the target to the image also changes, and there is an error between the tracking window and the actual target. In this paper, we build a scale pyramid of the current target in the original KCF algorithm. The template will carry out correlation filtering with each layer of images in the multi-scale pyramid and judge the scaling degree of the current target size according to the maximum filtering response value.

**Figure 2:**
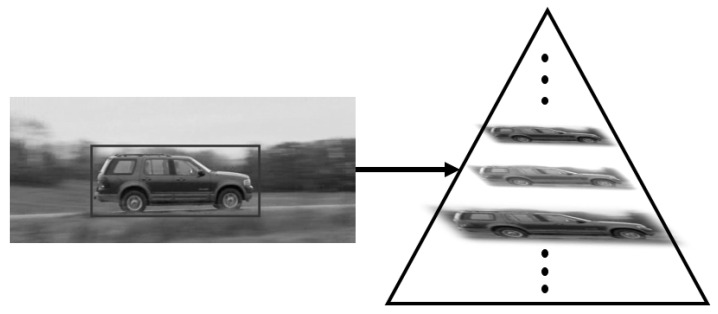
Construction of a multi-scale pyramid.

Assuming that ε is the scale factor of the target size of the current frame compared to the previous frame and $L_{t-1}$ is the target image of the previous frame, ε can be obtained by the following optimization formula:

(9) $$\max_{\varepsilon }C\left(T_{t-1},\varepsilon L_{t-1}\right)$$

where C(x,y) represents the correlation filtering results of *x* and *y* and *T* represents the tracking template. Since the correlation filter requires the same-sized input images, the scaled target images in the multi-scale pyramid need to be restored to the image with the same size as the previous frame through the resize operation. Meanwhile, the size of the patch window in KCF should also be scaled according to the scale factor ε determined in Formula (6). The construction process of the scale pyramid is shown in Figure 2.

### 3.5. Adaptive Template Updater Based on the Mean of Cumulative Maximum Response Values

**Figure 3:**
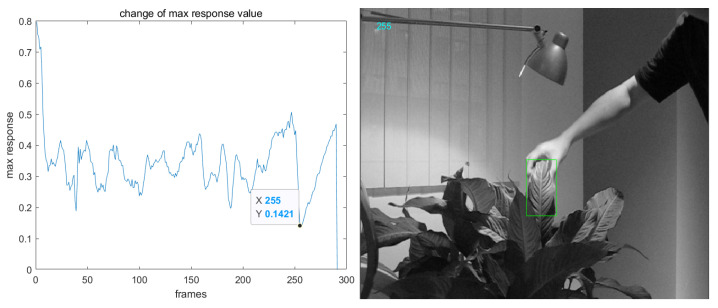
Left: the change in the maximum response value of the correlation filter; right: 255th frame of ‘coke’ sequence in OTB datasets.

The problem of target occlusion can easily make the template of the tracker blurred and lead to tracking failure. In the tracking process of a rigid target, the occlusion of a target can be judged according to the maximum response value of the correlation filter. The change in the maximum response value of the correlation filter in the original KCF tracking process is shown in Figure 3. As can be seen from the figure, when the target is in the normal tracking state, the maximum response value floats around an average value. When the target is occluded, the maximum response value is obviously lower than the mean. Therefore, this paper proposes to judge whether occlusion occurs according to the cumulative maximum response mean value and stops updating the tracker template if occlusion occurs.

Based on the above, we propose the criterion of the Mean of Cumulative Maximum Response Values (MCMRV), and the KCF tracker will implement adaptive template updates according to the MCMRV criteria. The maximum response value $m_{i}\left(i=1,2,\ldots ,t\right)$ is accumulated after *t* frames accumulation to obtain the cumulative value:

(10) $$C_{t}=\sum_{i=1}^{t}m_{i}$$

Therefore, the update threshold is:

(11) $$\beta_{t}=\theta \cdot \frac{C_{t}}{t}$$

The updating of the template parameter $\hat{a}$ and the sample parameter *X* based on MCMRV criteria yields the following expression, where θ indicates the allowed floating range:

(12) $$\left\{\begin{array}{c} \left\{\begin{array}{c} {\hat{a}}_{t+1}=\left(1-\mu \right){\hat{a}}_{t}+\mu {\hat{a}}_{t}\\ X_{t+1}=\left(1-\mu \right)X_{t}+\mu X_{t} \end{array}\right.,\;m_{t+1}\geq \beta_{t}\\ \left\{\begin{array}{c} {\hat{a}}_{t+1}={\hat{a}}_{t}\\ X_{t+1}=X_{t} \end{array},\right.\;m_{t+1}<\beta_{t} \end{array}\right.$$

In this paper, MCMRV is initialized by assuming that the tracker is in a normal trace state for γ frames after the trace begins, and the tracker template is updated according to Formula (5). When the number of frames is greater than γ, the MCMRV criterion is enabled for adaptive updating of the tracker template, according to Formula (7). The implementation of the adaptive multi-scale KCF based on MCMRV criterion (MMKCF) is shown in Algorithm 1.
**Algorithm 1:** MMKCF**Input:** 
The video frame, $I_{t}$; Initial bounding box of the target, bb;**Output:**
 The target position predicted by the tracker, *p*;  1:**while**$I_{t}$**do**  2:    **if** t==1 **then**  3:        $\left[{\hat{a}}_{0},X_{0}\right]=initialize\left(I_{1},bb\right)$  4:    **else**  5:        **if** 1<t≤γ **then**  6:           **for** $i=size\left(I_{t}\right)\times \;\left(1-\varepsilon \right)$ to $\;\;size\left(I_{t}\right)\times \left(1+\varepsilon \right)$ **do**  7:               $\left[max\_response\left(i\right),p\left(i\right)\right]=kernel\_correlation\_filter\left(I_{t},{\hat{a}}_{t},X_{t}\right)$  8:           **end for**  9:           $\left\{\begin{array}{c} {\hat{a}}_{t+1}=\left(1-\mu \right){\hat{a}}_{t}+\mu {\hat{a}}_{t}\\ X_{t+1}=\left(1-\mu \right)X_{t}+\mu X_{t} \end{array}\right.$10:           $C+=max\left(max\_response\right)$11:        **else**12:           **for** $i=size\left(I_{t}\right)*\;\left(1-\varepsilon \right):\;size\left(I_{t}\right)*\left(1+\varepsilon \right)$ **do**13:               $\left[max\_response\left(i\right),p\left(i\right)\right]=kernel\_correlation\_filter\left(I_{t},{\hat{a}}_{t},X_{t}\right)$14:           **end for**15:           **if** $max\left(max\_response\right)\leq \left(\beta_{t}=\theta \cdot \frac{C_{t}}{t}\right)$ **then**16:               $\;\left\{\begin{array}{c} {\hat{a}}_{t+1}={\hat{a}}_{t}\\ X_{t+1}=X_{t} \end{array}\right.$17:           **else**18:               $\left\{\begin{array}{c} {\hat{a}}_{t+1}=\left(1-\mu \right){\hat{a}}_{t}+\mu {\hat{a}}_{t}\\ X_{t+1}=\left(1-\mu \right)X_{t}+\mu X_{t} \end{array}\right.$19:               $C+=max\left(max\_response\right)$20:               p=max(p)21:           **end if**22:        **end if**23:    **end if**24:**end while**

## 4. Experiments

### 4.1. Datasets and Evaluate Metrics

In this section, we test and compare the performance of tracking algorithms on standard video database OTB-2013 [31] and OTB-2015 [32] datasets. The OTB-2013 dataset has 51 different video sequences, while the OTB-2015 dataset has 100 different video sequences, which all contain various attributes of targets, such as rigid target, non-rigid target, scale change target, fast motion target and so on. In particular, in order to analyze the tracking performance of the proposed algorithm for rigid targets, we focus on six typical video sequences in the dataset.

The main evaluation indicators are Frames Per Second (FPS) and One-Pass Evaluation (OPE) [31], which run trackers throughout a test sequence with initialization from the ground truth position in the first frame and report the average precision or success rate. Precision measures the Euclidean distance between the center point of the prediction box and the center point of the Ground Truth box. That is, their Euclidean distances are considered successful tracking if they are within the given threshold. Success rate is calculated as the bounding box overlap. Given the tracked bounding box $r_{t}$ and the ground truth bounding box $r_{a}$, the overlap score is defined as $S=\frac{|r_{t}\cap r_{a}|}{|r_{t}\cup r_{a}|}$, where ∩ and ∪ represent the intersection and union of two regions, respectively, and |•| denotes the number of pixels in the region.

### 4.2. Experimental Setup

In our approach, we assume that the scale change in the target between adjacent frames will not exceed 5%, so we set ε to 0.05. The update factor of the template μ is set to 0.02, the allowed floating range θ is set to 0.5 and γ is set to 50. These parameters will remain unchanged in the following experiments.

The BACF, SRDCF-decon, DSST, ECO-HC [33] and LADCF [34] tracking algorithms based on KCF were selected as the performance index comparison, and all the algorithms were run on an Intel Core I5-7200U CPU. Table 1 summarizes the differences between these trackers. The performance evaluation index is the tracking precision (20 px) of the sequences.

### 4.3. Experiment Results

On the basis of KCF, MMKCF combines a simple three-layer scale pyramid, which can adapt to the change in target size accurately and ensure the real-time performance of the tracker. At the same time, the adaptive template updater based on MCMRV criterion enables the tracker to deal with the problem of occlusion for a rigid target effectively.

#### 4.3.1. Experiments for Rigid Target on Selected Video Sequences

**Figure 4:**
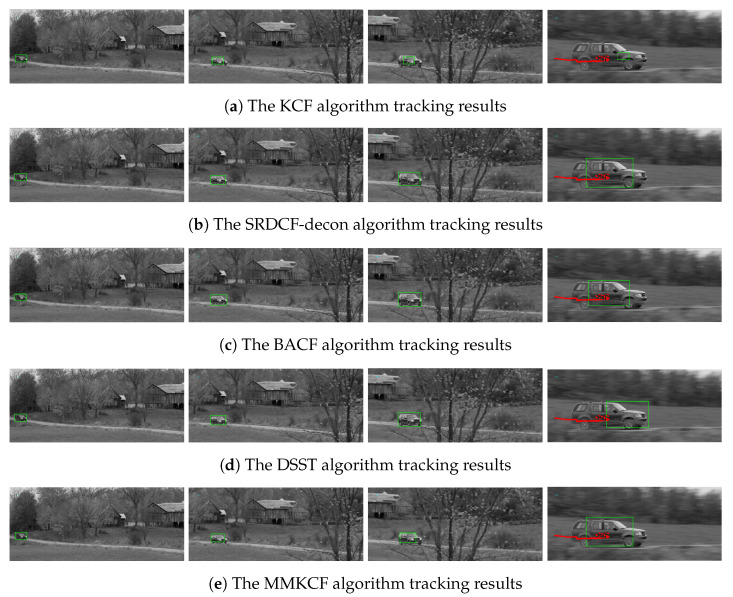
Tracking effect comparison of KCF, SRDCF-decon, BACF, DSST, and our MMKCF on the Car-scale, as shown in (**a**–**e**), respectively. These frames are 10, 100, 150 and 220, respectively. The red dot • represents the motion of the target center.

**Figure 5:**
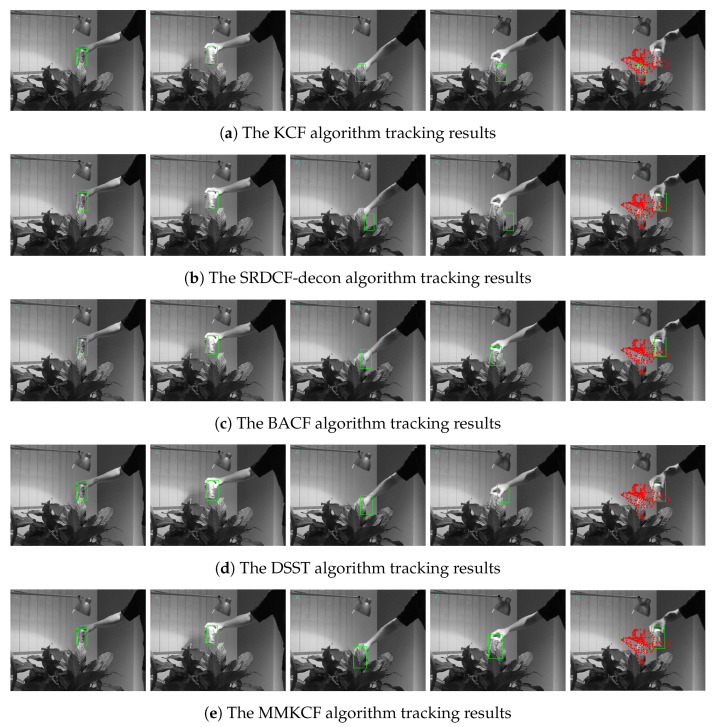
Tracking effect comparison of KCF, SRDCF-decon, BACF, DSST, and our MMKCF on Coke, as shown in (**a**–**e**), respectively. These frames are 10, 50, 260, 270 and 280, respectively. The red dot • represents the motion of the target center.

**Figure 6:**
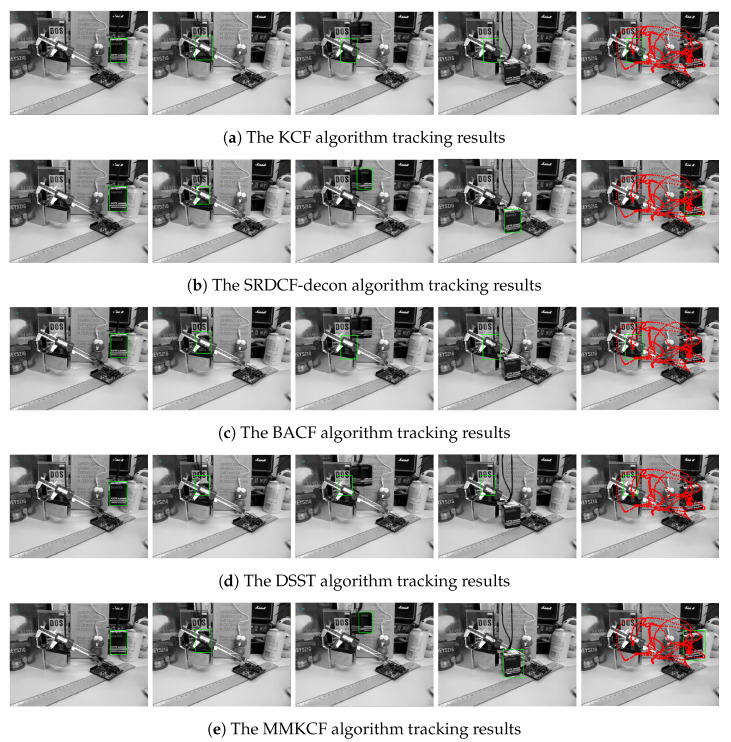
Tracking effect comparison of KCF, SRDCF-decon, BACF, DSST, and our MMKCF on Box, as shown in (**a**–**e**), respectively. These frames are 10, 460, 500, 800 and 900, respectively. Red dot • represents the motion of the target center.

The target template of KCF is the feature vectors extracted by HOG, but due to the fixed size of the target, it cannot adapt to the size change of the target, which will lead to a large amount of noise information introduced by the enlarged or reduced target in the matching template of the tracker during the update process, thus polluting the template and leading to tracking failure. Figure 4a,e show the comparison of the tracking effect between MMKCF and KCF on the Car-scale. In the video sequence, the size of the vehicle target keeps increasing, while the fixed-size target template can only be blinded by the progressively larger target. In contrast, MMKCF can quickly and accurately judge the size change of the target so that the target template adaptively changes with the target. As can be seen from a and b in Figure 4, SOTA algorithms, such as SRDCF-decon, BACF and LADCF, can well adapt to the scale change of the target, but their adaptation strategies are too complex to maintain the tracking speed, which will be mentioned in the later experimental results. The DSST algorithm (as shown in Figure 4d) adapts to the size change of the target, but its tracking box has deviated from the center position of the target in the tracking process. This is because the DSST algorithm does not handle target occlusion very well. When a vehicle is partially obscured by a tree trunk, the algorithm may suffer from template drift. Our algorithm adopts a stationary strategy when the target is occluded to avoid contamination of the template by the occluder. Therefore, it can still follow the target accurately when it is out of the occlusion. The main property of the Carscale sequence is scale variation, so the target is only briefly obscured, and the algorithms we tested were largely able to keep up with the target. In Figure 5 and Figure 6, experiment examples on the Coke and Box sequences will be shown, where the targets appear to be obscured for long periods of time.

The adaptive template updater of MMKCF can effectively deal with the problem of occluding rigid targets, as shown in Figure 5 and Figure 6. When the target occlusion occurs, the KCF algorithm cannot determine whether the occlusion occurs and continues to update the template of the tracker, resulting in extreme pollution of the template. When the target appears again, KCF cannot detect the target accurately and declares a trace failure, as shown in Figure 5a. MMKCF determines the occlusion of the target and stops updating the tracker template, retaining the position of the target detected the last time until the target appears in the field of view again. In Figure 5e, we find that the MMKCF algorithm can still accurately estimate the location of the target even when the target is blocked, and its performance is better than other SOTA algorithms when facing the occlusion problem. In Figure 5b–d, when the target is blocked, the algorithm cannot accurately predict the location of the target and mistakenly locks the target in hand.

Furthermore, if the target is shielded for a longer time, the tracking templates of KCF, BACF and DSST algorithms are easily contaminated, leading to tracking failure, as shown in Figure 6. In the second column of Figure 6, we can see the box target is obscured by the ruler and lighter. After about 40 frames of being occluded, the box target reappears in the field of view (third column of Figure 5), at which point the templates of the KCF, BACF and DSST algorithms are contaminated and the tracking is declared to have failed. Only our proposed algorithm and SRDCF-decon can successfully follow the target after it has been occluded for a long time. In this case, the MMKCF shows excellent performance, with the fastest processing speed in addition to successful target tracking.

**Figure 7:**
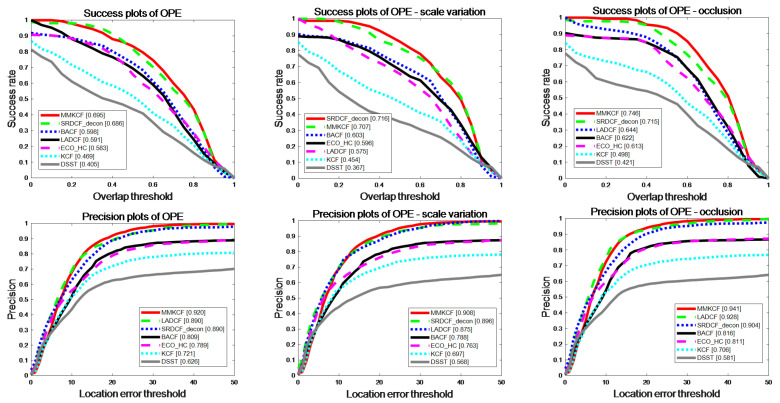
OPE comparison of our tracker and other 6 excellent trackers in terms of precision and success rate on the six selected video sequences.

**Figure 8:**
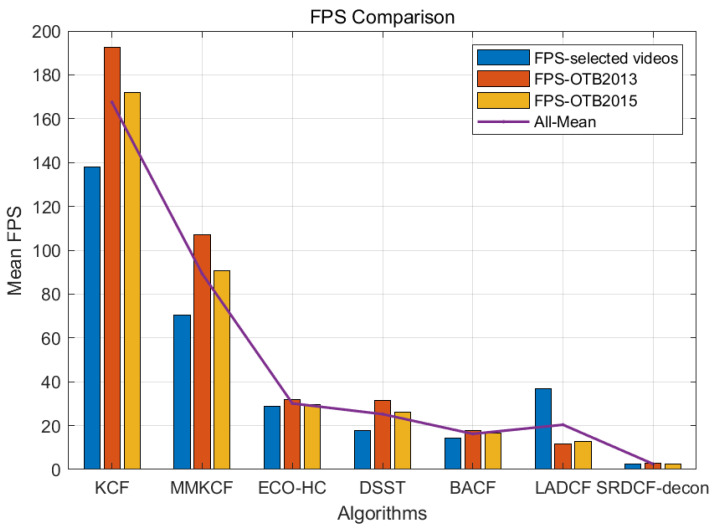
FPS comparison of our tracker and six other excellent trackers on the six selected video sequences, OTB2013 and OTB2015.

After our comprehensive evaluation on the selected datasets, MMKCF showed excellent performance in the tracking test for rigid targets. The results are shown in Table 2 and Table 3 and Figure 7 and Figure 8. It can be seen from Table 2 and Table 3 that MMKCF outperforms other SOTA algorithms in tracking performance. In comparison with KCF, BACF, ECO-HC, LADCF, SRDCF-Ddeon and DSST, MMKCF has higher tracking accuracy than 25.4%, 11.7%, 14.6%, 1.6%, 1.9% and 44.4%, respectively. Meanwhile, our algorithm is second only to KCF in FPS evaluation, with an average FPS of 72, far greater than other SOTA algorithms improved based on KCF. The other improved algorithms introduce more feature information and greatly improve the tracking accuracy, but none of them strike a good balance between speed and precision. OPE evaluation results show that the MMKCF algorithm is superior to other algorithms in precision and success rate, as shown in the success plots and precision plots of OPE in Figure 7. Although there is a small gap between MMKCF and LADCF in accuracy, it is obvious from the running speed that the calculation amount of the LADCF algorithm is much larger than that of MMKCF.

#### 4.3.2. Experiments on OTB2013 and OTB2015 Datasets

**Figure 9:**
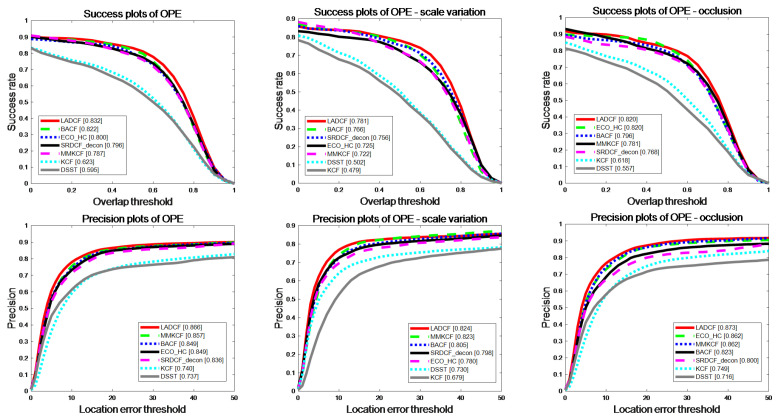
OPE comparison of our tracker and 6 other excellent trackers in terms of precision and success rate on OTB-2013.

**Figure 10:**
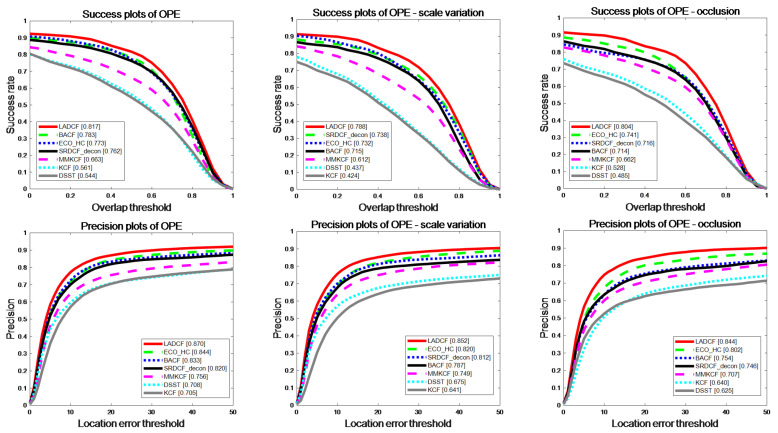
OPE comparison of our tracker and 6 other excellent trackers in terms of precision and success rate on OTB-2015.

Although OTB2013 and OTB2015 datasets contain a large number of non-rigid targets, such as humans and animals, which are not the research focus of this paper, OPE evaluation is still carried out on the complete OTB datasets to verify the generality and authenticity of our proposed algorithm, and the results are shown in Figure 8, Figure 9 and Figure 10. As can be seen from Figure 8, the MMKCF algorithm still maintains a high running speed on the complete OTB2013 and OTB2015 datasets, second only to the KCF algorithm. OPE evaluation shows that MMKCF’s tracking performance is significantly improved compared with KCF and DSST, and there is a small gap between MMKCF and other SOTA algorithms, as shown in Figure 9 and Figure 10. This is because we sacrificed some generalizability in exchange for performance and speed-up, and we achieved excellent results in experiments against rigid targets, which makes our algorithm convenient for military applications (against rigid targets, such as vehicles and ships). However, there is still room for improvement in our algorithm for multi-pose targets, such as humans and animals. In conclusion, experiments show that the MMKCF algorithm proposed by us is real and reliable in improving the tracking performance of rigid targets. Even when tracking non-rigid targets, the performance of the algorithm is significantly improved compared with the original KCF.

## 5. Conclusions

This paper improves the KCF algorithm mainly from two aspects: A simple multi-scale pyramid is integrated in KCF so that the tracker can adapt to the size change of the rigid target adaptively while ensuring the real-time requirement; the adaptive template updater based on MCMRV criterion enables KCF to deal with the problem of occlusion for a rigid target effectively. Experimental results show that our approach is effective and improves the precision and success rate of the tracking algorithm. Compared with other SOTA tracking algorithms based on kernel correlation filter, MMKCF can adapt to the scale change of the target well and deal with the problem of occlusion effectively while maintaining the high-speed processing ability. MMKCF is very suitable for embedded platforms with low power, small volume and limited computing power. Extensive experiments show that the proposed method is effective and real.

Although the proposed method can efficiently deal with the problem of target scale variation and occlusion, tracking accuracy is often reduced due to target illumination variation and motion blur in practical engineering. Meanwhile, solving the problem of targets that rotate or roll and cause tracking failure is also a worthwhile research priority. In future work, we will focus on exploring solutions to these problems and strive to promote the application of our academic research results in engineering projects.

## Figures and Tables

**Table 1:** The difference among eight trackers.

Name	Features	Scale Adaptive
MMKCF	HOG	YES
KCF	HOG	NO
BACF	HOG	YES
SRDCF_decon	HOG, CN	YES
ECO_HC	HOG, CN	YES
LADCF	HOG, CN	YES
DSST	HOG, CN	YES

**Table 2:** Precision comparison of experimental results on the six selected video sequences. The red marker indicates the best performance in the current sequence.

	Algo.	KCF	MMKCF	BACF	ECO-HC	LADCF	SRDCF	DSST
Seq.								
Car-scale		0.806	0.905	0.904	0.837	0.837	0.901	0.757
Coke		0.838	0.986	0.917	0.921	0.965	0.859	0.917
Vase		0.793	0.875	0.775	0.686	0.701	0.819	0.852
Lemming		0.495	0.923	0.871	0.910	0.907	0.912	0.429
Box		0.415	0.946	0.414	0.396	0.941	0.925	0.394
Liquor		0.976	0.794	0.974	0.985	0.984	0.910	0.404
MEAN		0.721	0.904	0.809	0.789	0.889	0.887	0.626

**Table 3:** FPS comparison of experimental results on the six selected video sequences. The red marker and bold indicate the best and the second best performance in the current data respectively.

	Algo.	KCF	MMKCF	BACF	ECO-HC	LADCF	SRDCF	DSST
Seq.								
Car-scale		217	**128**	15	27	16	4	45
Coke		122	**80**	16	29	11	2	15
Vase		155	**88**	14	28	16	2	18
Lemming		46	**35**	14	32	10	1	9
Box		32	**28**	13	30	11	2	7
Liquor		125	**71**	12	32	10	2	5
MEAN		116	**72**	14	30	12	2	17

## Data Availability

The data that support the findings of this study are available from the corresponding author upon reasonable request.

## References

[1] Ahmad T., Abbas A.M. (2021). Target tracking in wireless sensor networks. J. Comput. Sci. Technol..

[2] Song D. (2021). Image processing technology in american football teaching. Int. J. Electr. Eng. Educ..

[3] Lu H., Guna J., Dansereau D.G. (2017). Introduction to the special section on artificial intelligence and computer vision. Comput. Electr. Eng..

[4] Yilmaz A., Javed O., Shah M. (2006). Object tracking: A survey. ACM Comput. Surv. (CSUR).

[5] Chai X., Wu H., Gan Z., Zhang Y., Nixon K.W. (2020). An efficient visually meaningful image compression and encryption scheme based on compressive sensing and dynamic lsb embedding. Opt. Lasers Eng..

[6] Ding W., Wang A.C., Wu C., Guo H., Wang Z.L. (2018). Human-machine interfacing enabled by triboelectric Nanogenerators and Tribotronics. Adv. Mater Technol..

[7] Tian W., Zhang G., Alam B.Z., Liu A., Jia W., Xie M. (2018). A novel trust mechanism based on fog computing in sensor–cloud system. Future Gener. Comput. Syst..

[8] Li X., Zha Y.F., Zhang T.Z., Cui Z., Zuo W.M., Hou Z.Q., Wang H.Z. (2019). Survey of visual object tracking algorithms based on deep learning. J. Image Graph..

[9] Bolme D.S., Beveridge J.R., Draper B.A., Lui Y.M. Visual object tracking using adaptive correlation filters. Proceedings of the 2010 IEEE Computer Society Conference on Computer Vision and Pattern Recognition.

[10] Krizhevsky A., Sutskever I., Hinton G.E. (2017). ImageNet classification with deep convolutional neural networks. Commun. ACM.

[11] Fan H., Ling H. Sanet: Structure-aware network for visual tracking. Proceedings of the IEEE Conference on Computer Vision and Pattern Recognition Workshops.

[12] Henriques J.F., Caseiro R., Martins P., Batista J. (2014). High-speed tracking with kernelized correlation filters. IEEE Trans. Pattern Anal. Mach. Intell..

[13] Zhang J.H., Li P., Jin C.C., Zhang W.A., Liu S. (2019). A novel adaptive kalman filtering approach to human motion tracking with magnetic-inertial sensors. IEEE Trans. Ind. Electron..

[14] Bhat P.G., Subudhi B.N., Veerakumar T., Laxmi V., Gaur M.S. (2020). Multi-feature fusion in particle filter framework for visual tracking. IEEE Sens. J..

[15] Comaniciu D., Meer P. (2002). Mean shift: A robust approach toward feature space analysis. IEEE Trans. Pattern Anal. Mach. Intell..

[16] Hsia C.H., Liou Y.J., Chiang J.S. (2016). Directional prediction camshift algorithm based on adaptive search pattern for moving object tracking. J. Real-Time Image Proc..

[17] Henriques J.F., Caseiro R., Martins P., Batista J. (2012). Exploiting the circulant structure of tracking-by-detection with kernels. European Conference on Computer Vision.

[18] Danelljan M., Häger G., Khan F., Felsberg M. Accurate scale estimation for robust visual tracking. Proceedings of the British Machine Vision Conference.

[19] Danelljan M., Häger G., Khan F.S., Felsberg M. (2016). Discriminative scale space tracking. IEEE Trans. Pattern Anal. Mach. Intell..

[20] Danelljan M., Hager G., Shahbaz Khan F., Felsberg M. Learning spatially regularized correlation filters for visual tracking. Proceedings of the IEEE International Conference on Computer Vision.

[21] Kiani Galoogahi H., Fagg A., Lucey S. Learning background-aware correlation filters for visual tracking. Proceedings of the IEEE International Conference on Computer Vision.

[22] Simonyan K., Zisserman A. (2014). Very deep convolutional networks for large-scale image recognition. arXiv.

[23] He K., Zhang X., Ren S., Sun J. Deep residual learning for image recognition. Proceedings of the IEEE Conference on Computer Vision and Pattern Recognition.

[24] Redmon J., Divvala S., Girshick R., Farhadi A. You only look once: Unified, real-time object detection. Proceedings of the IEEE Conference on Computer Vision and Pattern Recognition.

[25] Goodfellow I., Pouget-Abadie J., Mirza M., Xu B., Warde-Farley D., Ozair S., Bengio Y. Generative adversarial nets. Proceedings of the Advances in Neural Information Processing Systems.

[26] Nam H., Han B. Learning Multi-Domain Convolutional Neural Networks for Visual Tracking. Proceedings of the IEEE Conference on Computer Vision and Pattern Recognition.

[27] Song Y., Ma C., Wu X., Gong L., Bao L., Zuo W., Shen C., Lau R.W.H., Yang M.H. Visual tracking via adversarial learning. Proceedings of the 2018 IEEE Conference on Computer Vision and Pattern Recognition.

[28] Bertinetto L., Valmadre J., Henriques J.F., Vedaldi A., Torr P.H. (2016). Fully-convolutional siamese networks for object tracking. European Conference on Computer Vision.

[29] Felzenszwalb P.F., Girshick R.B., McAllester D., Ramanan D. (2010). Object detection with discriminatively trained part-based models. IEEE Trans. Pattern Anal. Mach. Intell..

[30] Hong D., Balzano L., Fessler J.A. (2018). Asymptotic performance of PCA for high-dimensional heteroscedastic data. J. Multivar. Anal..

[31] Yi W., Lim J., Yang M.H. Online object tracking: A benchmark. Proceedings of the Computer Vision Pattern Recognition.

[32] Wu Y., Lim J., Yang M.H. (2015). Object tracking benchmark. IEEE Trans. Patt. Anal. Mach. Intell..

[33] Danelljan M., Bhat G., Shahbaz Khan F., Felsberg M. Eco: Efficient convolution operators for tracking. Proceedings of the IEEE Conference on Computer Vision and Pattern Recognition.

[34] Xu T., Feng Z.H., Wu X.J., Kittler J. (2019). Learning adaptive discriminative correlation filters via temporal consistency preserving spatial feature selection for robust visual object tracking. IEEE Trans. Image Process..

